# Probiotic growth in skin-like conditions

**DOI:** 10.3934/microbiol.2022027

**Published:** 2022-10-27

**Authors:** MP Lizardo, FK Tavaria

**Affiliations:** Centro de Biotecnologia e Química Fina–Laboratório Associado, Escola Superior de Biotecnologia, Universidade Católica Portuguesa/Porto, Rua Diogo Botelho, 1327, 4169-005 Porto, Portugal

**Keywords:** skin, probiotics, abiotic, UV radiation, fatty acids, NaCl, skin-like conditions

## Abstract

Although probiotics' main known effects are in the digestive system, over the last years several benefits that come from their topical use, have been investigated. Several studies have reported beneficial effects on different skin disorders, such as atopic dermatitis, acne, eczema, psoriasis, wound healing, skin aging and reactive skin. Their main action is assigned to the inhibition of skin colonization by pathogens. In this work, the growths of three probiotic strains were evaluated in the presence of abiotic factors similar to those found in skin, namely, UV radiation, temperature, pH, NaCl and fatty acids. *Lactobacillus rhamnosus* showed increased growth under the pH of 6, but no differences in its growth were found for the various NaCl concentrations tested. *Lactobacillus delbrueckii* increased the number of bacterial cells in 88.8% when grown in 10 mM NaCl concentration, while *Propioniferax innocua* showed increased growth at 45 °C. All tested probiotic bacteria were able to grow under skin-like conditions. However, *L. rhamnosus* was the probiotic that showed the best results. The results obtained in this study indicate that the used probiotics may be beneficial in the treatment of skin diseases, since they are able to successfully thrive in skin-like conditions.

## 1. Introduction

The main function of the skin is to act as a physical barrier and protect the body against pathogenic organisms or toxic substances [1]. It is constituted by three major layers, the dermis, the epidermis and the hypodermis, and it is continuously dynamic due to its protective function [2]. The epidermis is the main outer layer and is mostly constituted by keratinocytes (90–95%), responsible for skin color, texture and moisture [3]. Beneath the epidermis is the dermis, which provides vascular supply to the skin; and below the dermis is the hypodermis, which is mostly composed of adipose tissue and collagen [4].

Generally, the skin is a cold, acidic and dry environment. Due to these harsh conditions, just a limited number of microbial species can inhabit there; however, despite this inhospitable environment, it is estimated that approximately 1 billion bacteria are present per square centimeter of skin [5]. The microbiota is generally divided into two groups: resident microbes, which are routinely found in the skin and are often considered commensal, and transient microorganisms, which come from exogenous sources and can persist in the skin for hours or days but not permanently [6].

Healthy human skin typically contains a mixture of Gram-positive and Gram-negative bacteria of different phyla. Grice *et al*. (2009), using 16S ribosomal RNA (rRNA) gene phylotyping, characterized the skin microbiomes of 10 healthy humans in 20 different skin locations. Nineteen different phyla were detected, with *Actinobacteria* being predominant, followed by (in descending order) *Firmicutes*, *Proteobacteria* and *Bacteroidetes*. *Propionibacteria*, *Corynebacteria* and *Staphylococci* were the most prevalent genera [7].

Skin microbiota can be influenced by diverse host factors, such as genetics, skin site, sex, immune status and skin disease [8]. However, not only the physical skin structure turns skin into a complex environment; the environmental factors that the skin is exposed to also contribute to it, since the skin is one of the most unprotected organs in the human body [9]. Therefore, dysbiosis is a common issue resulting in different skin conditions, which can be ameliorated through the use of probiotics, as described in recent reviews [10].

The UV radiation that reaches the earth is composed of UVA (400–320 nm) and UVB (320–290 nm) rays. The longer wavelengths have lower energy but can penetrate deeply into the dermis [11]. The radiation varies according to the season, time of the day, latitude, altitude and O_3_ column [12]. Depending on the intensity and time of exposure, the radiation can alter the genomic composition of microbes and, therefore, the skin microbiome. Nevertheless, some bacteria and fungi have demonstrated tolerance to this radiation in parts of their life cycles [13]. Exposure to solar UV radiation can lead to epidermal oxidative stress by inducing the generation of reactive oxygen species (ROS); these, on the skin surface, modify gene and protein structures and functions [14],[15]. UV radiation is also involved in skin freckling, wrinkling and photoallergic response and is known as the primary cause of skin cancer [13],[14]. Probiotics can be useful for preventing epidermal oxidative stress by the topical route or via ingestion [15].

Skin pH is a factor affecting the barrier function, homeostasis, stratum corneum (SC) integrity and cohesion and antimicrobial defense mechanisms, and it is a factor controlling the cutaneous microbiota, contributing to a good skin condition [15],[16]. The normal skin surface pH is acidic, varying from 4 to 6, while the internal pH is close to neutral [16]. This acidic pH may be due to components produced by a passive mechanism; among these are lactic acid, free fatty acids, cholesterol sulfate, urocanic acid and pyrrolidone carboxylic acid [17]. An increase of pH in this structure is accompanied by the perturbation of cutaneous permeability barrier homeostasis and associated with several epidermal disorders, like eczema, atopic dermatitis and seborrheic dermatitis. In order to modulate the cutaneous pH, some probiotics like lactic acid bacteria (LAB) can be used, as they help in lowering the pH [15].

Sweat reduces the bacterial load in healthy individuals, and its production rate directly affects the concentration of salt in the skin. The salt concentration in sweat is estimated to be between 18 mM and 60 mM [18]. It has been shown that excess NaCl can affect the innate immune system by modulating T cell response, in particular, increasing TH17 cell differentiation [19]. Also, NaCl is an ionic checkpoint for human type 2 immunity (an adaptive immune response with distinct cellular and cytokine selection, essential for host resistance against helminthic (worm) infections, although it can cause atopic reactions when dysregulated), with potential clinical relevance for atopic dermatitis [20].

The skin's surface temperature is an essential physiological parameter since it reflects the state of heat exchange between the human body and the thermal environment [21]. The temperature effect on microorganisms has been studied, showing that with higher temperatures, the numbers increase [22]. Body temperature fluctuates with a person's circadian rhythm, with daily activities (eating, exercising and sleeping) and even with gender [21].

A complex mixture of lipids can be found on the surface of the skin, deriving from the sebum. This mixture contains triacylglycerols, diacylglycerols, non-esterified fatty acids, wax esters, squalene and cholesterol esters. Ten percent of the mass of the SC is constituted of barrier lipids secreted by the keratinocytes; that barrier consists of 50% ceramides, 25% cholesterol and 15% non-esterified fatty acids [2]. In order to maintain adequate barrier homeostasis, the three lipidic components must be present. Free fatty acids are essential for the development of the epidermal barrier, serving as building blocks for more complex lipids like ceramides, contributing to the lipid matrix structure [23]. The sebum consists mainly of squalene, esters of glycerol, wax and cholesterol, as well as fatty acids. Fatty acids together with triglycerides account for 57.5% [24], the predominant portion. The major fatty acid components in the human epidermis are palmitic acid, stearic acid, palmitoleic acid, (all-cis)-11,14,17-eicosatrienoic acid and linoleic acid [25]. Linoleic acid, one of the essential fatty acids, is involved in the establishment and maintenance of the epidermal water barrier, the disturbance of which is one of the epidermal abnormalities of cutaneous essential fatty acid deficiency [26].

In this study, we evaluated the effects of some abiotic factors (UV exposure, pH, NaCl concentration, temperature and lipids) on skin microorganisms, and we concluded that probiotics can potentially be used to modulate the skin microbiome, since they are able to thrive in skin-like conditions.

## 2. Materials and methods

### 2.1. Bacterial strains and culture conditions

The probiotic strains used in this study were *Lactobacillus delbrueckii* subsp. *bulgaricus* 20081, *Lactobacillus rhamnosus* 20021 and *Propioniferax innocua* 8251 (DSMZ, Braunschweig, Germany). The strains were obtained as frozen stocks at −78 °C, then cultured on de Man, Rogosa and Sharpe broth (MRS, Biokar diagnostics, Allone, France) media and incubated at 37 °C for 24 h.

### 2.2. Growth curves

Standard bacterial growth curves were made for all bacteria under study, by measuring the optical density and plating onto solid media to calculate the respective CFUs (colony forming unit). Overnight cultures were inoculated in MRS broth in 1:100 proportions, and the inoculum was stored at 37 °C during the time of the procedure. Then, the optical density was measured at 600 nm, in a Nicolet Evolution 100 UV-Vis apparatus (Thermo Electron Corporation, Waltham, MA, USA), at periodic intervals of 1 hour up to 12 hours and up to 24 hours. Afterward, dilutions in Ringer's solution were made and plated (100 µL) onto MRS in duplicate. The colonies were counted approximately 24 h after incubation at 37 °C.

### 2.3. Probiotic bacterial growth in skin-like abiotic factors

To evaluate the probiotic survival when exposed to sunlight, an assay with the UV radiation of a laminar flow chamber was done. For that, one milliliter of bacteria after an overnight culture was transferred to plates with 9 mL of MRS media, in triplicate for each time, per bacteria. The plates were then left on the chamber irradiated by the UV lamp (Philips TUV T8 30 W, with wavelength peak at 253.7 nm). The triplicates were taken out and plated at 30 min intervals during three hours. The plating was done in MRS agar media for all dilutions. After approximately 24 h of incubation at 37 °C, the colonies were counted.

### 2.4. pH and NaCl concentration

Different solutions were made to ascertain the effects of pH and NaCl concentration on the bacterial growth in conditions close to those present on the skin. For different pH values, using NaOH and HCl solutions, the pH of the media (MRS) was changed to 3, 4, 5, 6 and 7 (Crison Micro pH 2002, Barcelona, Spain). For [NaCl], MRS with different NaCl concentrations was prepared. The tested NaCl concentrations were 10 mM, 20 mM, 40 mM, 60 mM and 80 mM.

Then, in both cases, the different bacteria strains after overnight culture were added (100 µL) to the different modified media (900 µL) in an Eppendorf tube, and 100 µL were transferred to a Thermo Scientific 96-well microplate. The microplate containing the bacterial cultures was incubated at 37 °C for 24 hours, during which optical density (600 nm) of the culture was measured at 3 h intervals in a Multiskan Sky 6.0.2 apparatus (Thermo Fisher, Waltham, MA, USA). The control included 100 µL of bacteria and 900 µL of MRS broth only.

### 2.5. Temperature

Growth at different temperatures was also assessed, considering the range that skin can be exposed to. So, temperatures of 18 °C, 25 °C, 37 °C and 45 °C were chosen to evaluate growth. In four distinct days, after overnight culture in MRS broth, the different probiotic strains (100 µL) were added to 900 µL of fresh media in Eppendorf tubes. After mixing, 100 µL was transferred to a 96-well microplate. Then, the different microplates were incubated at 18 °C, 25 °C, 37 °C and 45 °C for 24 hours, with optical density (600 nm) reads at periods of 3 h in the same equipment referred to above. Since the equipment is not able to decrease the temperature, the 18 °C had a degree of error of ± 3 °C.

### 2.6. Fatty acids

Since the skin's structure comprises lipids such as fatty acids, their effect on bacterial growth was also tested. The fatty acids were diluted in MRS broth media to obtain similar percentages to those found on the skin. The ones that were used in this assay were palmitic acid (9%) and linoleic acid (6%), making a total of 15% of fatty acids together (mix). The sterile MRS broth was added to the lipidic content in Eppendorf tubes, and after mixing, these were filtered with 0.20 µm filters to another tube. Then, solutions with a concentration of 1/10 of bacteria were added. Next, 50 µL was transferred to a 96-well microplate and incubated at 37 °C for 24 h, reading the optical density (600 nm) of the culture at 3 h intervals. The control was done with bacteria and MRS broth only.

### 2.7. Statistical analysis

The data analysis was made through IBM SPSS (IBM, New York, USA) using one-way ANOVA. When necessary, multiple comparisons with the post-hoc Tukey test were performed. The significance level considered was 0.05.

## 3. Results

The growth curves are essential to determine the time after inoculation at which each bacterium enters the stationary growth phase, which may be defined as the phase at which the total cell absorbance ceases to increase. It becomes even more important since some probiotics might produce bacteriocins in their exponential and stationary phases, reaching a peak in the last one [4]. Taking this into account, growth curves of colony-forming units (CFU) and optical density at 600 nm (wavelength usually used in bacterial analysis) were made for all bacteria under study.

**Figure 1.**
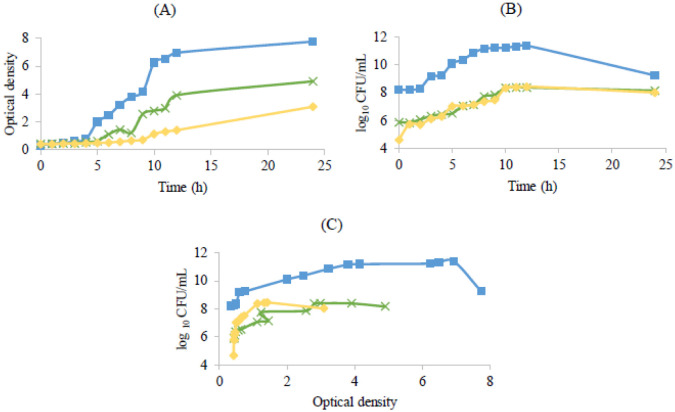
Bacterial growth curves of the probiotic strains used in the assay. Optical density at 600 nm versus time (A), log_10_ colony-forming units per milliliter versus time (B) and log_10_ CFU/mL versus optical density at 600 nm (C). *Lactobacillus rhamnosus* (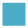), *Lactobacillus delbrueckii* subsp. *bulgaricus* (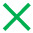) and *Propioniferax innocua* (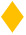).

Figure 1 shows these growth patterns in aerobic incubating conditions.

### 3.1. Bacterial growth in skin-like abiotic factors

To establish the patterns of growth of probiotic bacteria, when topically applied to the skin, the three probiotic bacteria were submitted to different abiotic conditions similar to those found in our skin, as mentioned previously.

### 3.2. UV radiation

UV radiation is the major source of mutagenic and lethal effects by solar radiation, and in nature, bacterial cells are subject to DNA damage from solar radiation exposure [27],[14]. The probiotic bacterial strains were exposed to UVC radiation, with a wavelength in the range of 100–250 nm, for 3 h [28].

**Figure 2.**
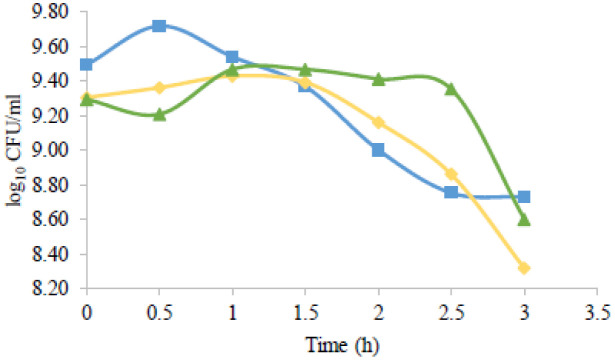
Bacterial growth curves after exposure to UV radiation: *Lactobacillus rhamnosus* (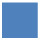), *Lactobacillus delbrueckii* subsp. *bulgaricus* (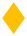) and *Propioniferax innocua* (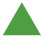).

The growth curves obtained are shown in Figure 2, where it is possible to see that at the end of the time of the procedure, a decrease of approximately one logarithmic unit in all three bacteria was observed.

### 3.3. pH

**Figure 3.**
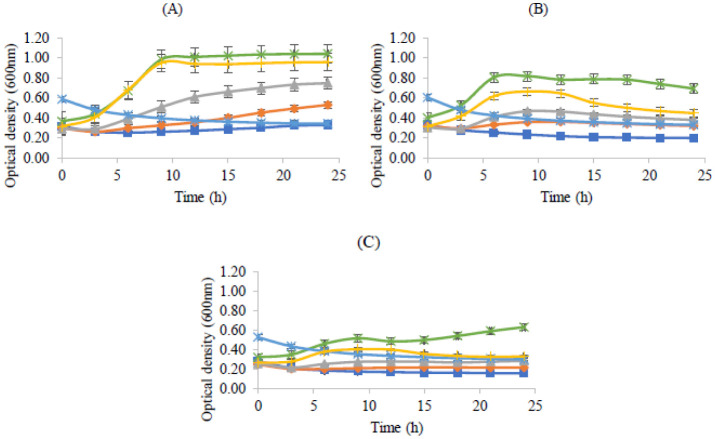
Bacterial growth (measured by optical density at 600 nm) in MRS broth with pH 3 (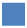), pH 4 (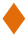), pH 5 (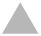), pH 6 (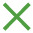), pH 7 (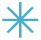) and control (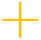) during 24 hours. (A) *Lactobacillus rhamnosus,* (B) *Lactobacillus delbrueckii* subsp. *bulgaricus* and (C) *Propioniferax innocua*. Error bars are ± standard deviation.

It is well known that pH influences the incidence and distribution of microorganisms, including bacteria [29]. The results obtained in this study for pH are shown in Figure 3, and the control of the assay was done with MRS broth media with no pH alteration, which according to the provider is 6.2. The choice of pH values was based on the reported skin pH values of 4–6, as indicated previously [14],[15].

### 3.4. NaCl concentration

**Figure 4.**
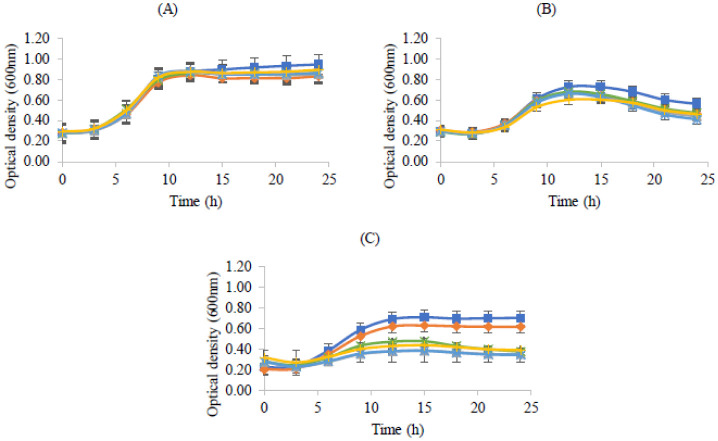
Bacterial growth (measured by optical density at 600 nm) in MRS broth with [NaCl] of 10 mM (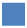), 20 mM (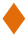), 40 mM (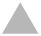), 60 mM (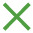), 80 mM (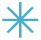) and control (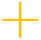) during 24 hours. (A) *Lactobacillus rhamnosus,* (B) *Lactobacillus delbrueckii* subsp. *bulgaricus* and (C) *Propioniferax innocua*. Error bars are ± standard deviation.

NaCl has been related to bacterial growth, being able to inhibit harmful bacteria. The results concerning the influence of different NaCl concentrations on the optical densities of the studied probiotic strains can be found in Figure 4. The NaCl concentration values used are in agreement with the values reported for sweat salt concentration (between 18 and 60 mM) [18]. According to the obtained results, the tested concentrations of sodium chloride do not exert relevant growth changes on both *Lactobacillus* strains. For the different NaCl concentrations, identical OD values were obtained. However, *P*. *innocua* showed significant growth with 10 mM NaCl and less growth at 80 mM of NaCl.

### 3.5. Temperature

**Figure 5.**
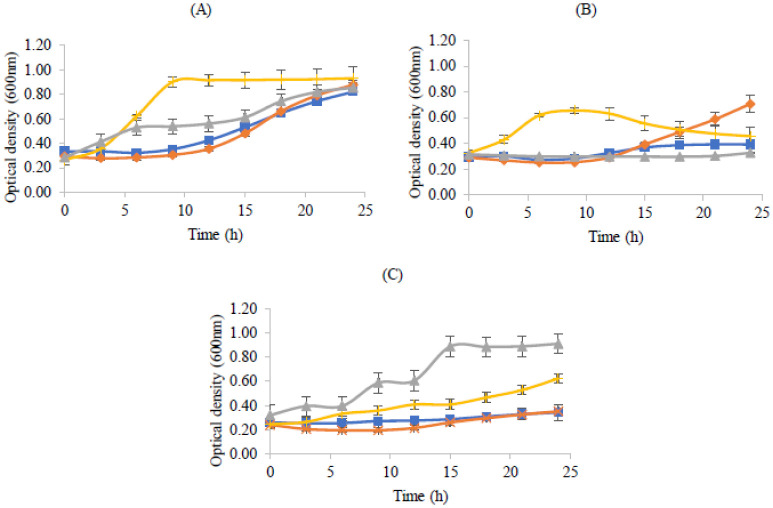
Bacterial growth (measured by optical density at 600 nm) in MRS broth with different incubation temperatures: 18 °C (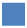), 25 °C (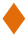), 45 °C (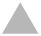) and control at 37 °C (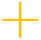) during 24 hours. (A) *Lactobacillus rhamnosus,* (B) *Lactobacillus delbrueckii* subsp. *bulgaricus* and (C) *Propioniferax innocua*. Error bars are ± standard deviation.

Temperature is a cardinal factor in the control of the growth rates of microbial populations [30]. The temperatures that our skin can be exposed to influence the probiotic growth rate over time. This was analyzed, and the obtained results are presented in Figure 5. The temperatures used were chosen according to temperature variation in the toe [30],[31].

Analyzing the results, it can be noted that, as expected, 37 °C was the temperature with more accentuated growth of the bacterium *Lactobacillus rhamnosus*.

### 3.6. Fatty acids

**Figure 6.**
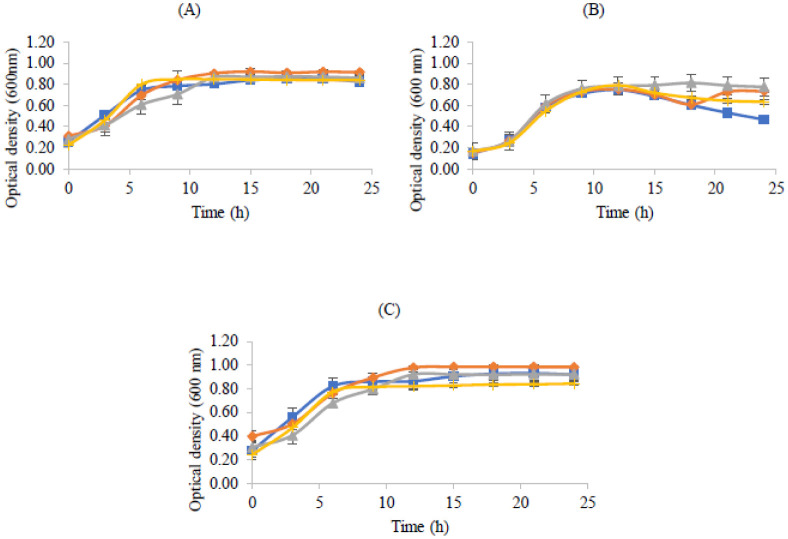
Bacterial growth (measured by optical density at 600 nm) in MRS broth with the addition of fatty acids: linoleic acid 6% (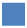), palmitic acid 9% (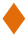), linoleic acid plus palmitic acid 15% (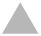) and control (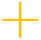) during 24 hours. (A) *Lactobacillus rhamnosus,* (B) *Lactobacillus delbrueckii* subsp. *bulgaricus* and (C) *Propioniferax innocua*. Error bars are ± standard deviation.

The influences of typical skin concentrations of fatty acids in the growths of the probiotics were explored, resulting in the graphs presented in Figure 6. The concentrations used were based on the percentages of fatty acids in human skin as reported by Kendall *et al*. (2017). The used concentrations of fatty acids did not affect the growth of the probiotics tested, since all three strains do not present significative differences in the OD curves.

## 4. Discussion

*L*. *rhamnosus* showed higher growth, as expressed by the viable numbers as well as optical density (Figure 1), when compared to the other two bacteria under study, probably related to its ability to grow under aerobic conditions [32], when compared to the majority of the strains from the *L. casei* group. According to these authors and other previous studies [33], when exposed to aerobic and respiratory growth conditions, *L. rhamnosus* increased biomass because of extra ATP generation and increased external pH due to the conversion of pyruvate into acetate. *Lactobacillus delbrueckii* subsp. *bulgaricus* is an aerotolerant anaerobe, not requiring strict anaerobic growth conditions and tolerating oxygen to concentrations present in the air [33]. Although it does not use O_2_ in its metabolism, the presence of this element can influence its physiology, probably promoting an early entry into the stationary phase, due to the oxidative stress caused by an excessive liberation of hydrogen peroxide, mostly due to NADH oxidase, an enzyme involved in the elimination of dissolved oxygen [33].

Figure 2 suggests that the probiotic strains under study can withstand UV radiation. UVC radiation is the most harmful to genetic integrity since the maximum absorption spectrum of DNA is 260 nm, and it is thus able to induce apoptosis or cell cycle arrest [34]. For that reason, it is used in most bactericidal studies, especially the wavelength of 254 nm [12].

Regarding the behavior of bacteria when exposed to different pH values, *Lactobacillus rhamnosus* (Figure 3A), unsurprisingly, grew better at pH 6 and showed less growth at pH 3. This result agrees with the literature [35], where optimal growth at pH values between 4.5 and 6.4 is reported. *Lactobacillus delbrueckii* subsp. *bulgaricus* (Figure 3B) also presented accentuated growth at pH 6; these results are expected since this bacterium has an optimal growth pH of 5.8 [36]. *Propioniferax innocua* (Figure 3C) also grew well at pH 6. Although the literature refers to an optimal pH of 7, in this study, this was not the best pH value for this probiotic to grow. Nonetheless, it consistently displayed lower optical density values than *Lactobacillus* strains. A decrease in OD, as shown by all tested bacteria at pH 7, could be related to cell lysis [37]. The control (pH 6.2) was revealed to be the second best pH value where all three bacteria grew better, although with values remarkably close to the results obtained for pH 6. These results suggest that all three probiotic strains tested in this assay can grow at skin-like pH, which has been found to be between 4 and 6 [16].

The obtained results for growth at different concentrations of NaCl are in agreement with earlier documented studies. Prasad and colleagues (2003) reported that *L*. *rhamnosus* exposed to 0.3 M of NaCl did not show any growth alterations, whereas at higher salt concentrations (0.4 M to 0.7 M), the growth rate decreased [38]. On the other hand, Chun *et al*. (2012) [37] reported that a NaCl concentration of 0.2 M inhibited the growth of *L*. *delbrueckii*. However, the mentioned studies used NaCl concentration values much higher than those tested in the present study. In the current study, *P*. *innocua*'s growth was not affected, since the NaCl concentrations used were much lower than those in Yokota's (1994) study, where he reported growth for *P*. *innocua* at 7.5% but not at 10% NaCl. Considering this, the salt concentration in sweat will not constitute a problem for the growth of probiotic bacteria since it is estimated to be between 18 and 60 mM [18].

As mesophilic bacteria, these strains have optimal growth temperatures of 37 °C, achieving high cell yields at this temperature. Nevertheless, it is reported that, depending on the strain, *L*. *rhamnosus* may grow at temperatures lower than 15 °C and higher than 40 °C [35].

*L*. *delbrueckii* showed higher OD values at 37 °C and 25 °C. Although in the literature it is referred to that the optimal temperature for this probiotic is between 42 °C and 55 °C [36], in this work, for a temperature within this range (45 °C), it exhibited lower OD values.

*P*. *innocua* grows better at 45 °C, whereas 37 °C has been pointed to as the optimum growth temperature [41]. While according to Yokota (2015) [39], this bacterium is capable of growth between 10 and 40 °C, in the current experiment the growth at 18 and 25 °C was very limited. These results demonstrate that probiotics may grow within the range of skin temperature variations.

Despite the growths of the studied probiotics in the presence of fatty acids not being well documented, this study shows no significant differences in the growth patterns in the presence of linoleic and palmitic acids, whether isolated or in combination (Figure 6). Also, it is important to note that MRS medium contains 0.1% Tween 80, which is a water-soluble derivative of oleic acid [42]. Since this is considered the optimal medium for Lactic Acid Bacteria growth (and the concentration of Tween 80 is low), it might be assumed that linoleic (6%) and palmitic acids (9%) did not affect the growth of the studied bacteria.

## 5. Conclusion

*Lactobacillus rhamnosus*, *Lactobacillus delbrueckii* subsp. *Bulgaricus* and *Propioniferax innocua*, as probiotics, were able to successfully grow in skin-like conditions. This growth was more relevant for *L*. *rhamnosus*, which showed increased growth at pH 6, with no major differences in growth for the various NaCl concentrations under study. Additionally, *L*. *delbrueckii* showed increased growth (88.8%) in the presence of 10 mM NaCl, while *P*. *innocua* had significantly increased growth at 45 °C. All tested bacteria showed similar growth trends in the presence of both fatty acids under study. Although UV radiation decreased the bacterial viable counts of *L*. *rhamnosus* (8.0%), *L*. *delbrueckii* (10.0%) and *P*. *innocua* (7.4%), these results were only apparent after three hours of exposure. Although each bacteria had different growth trends according to the abiotic factor evaluated, it is safe to say that under the tested conditions, probiotics can persist and therefore may be able to successfully colonize the skin.
